# Stamping Fabrication of Flexible Planar Micro‐Supercapacitors Using Porous Graphene Inks

**DOI:** 10.1002/advs.202001561

**Published:** 2020-07-27

**Authors:** Fei Li, Jiang Qu, Yang Li, Jinhui Wang, Minshen Zhu, Lixiang Liu, Jin Ge, Shengkai Duan, Tianming Li, Vineeth Kumar Bandari, Ming Huang, Feng Zhu, Oliver G. Schmidt

**Affiliations:** ^1^ Material Systems for Nanoelectronics Chemnitz University of Technology Chemnitz 09107 Germany; ^2^ Center for Materials Architectures and Integration of Nanomembranes (MAIN) Chemnitz University of Technology Chemnitz 09126 Germany; ^3^ Institute for Integrative Nanosciences Leibniz IFW Dresden Dresden 01069 Germany; ^4^ School of Materials Science and Engineering Ulsan National Institute of Science and Technology (UNIST) Ulsan 44919 Republic of Korea; ^5^ State Key Laboratory of Polymer Physics and Chemistry Changchun Institute of Applied Chemistry Chinese Academy of Sciences Changchun 130022 P. R. China; ^6^ School of Science Dresden University of Technology Dresden 01062 Germany

**Keywords:** areal energy density, graphene inks, micro‐supercapacitors, stamping

## Abstract

High performance, flexibility, safety, and robust integration for micro‐supercapacitors (MSCs) are of immense interest for the urgent demand for miniaturized, smart energy‐storage devices. However, repetitive photolithography processes in the fabrication of on‐chip electronic components including various photoresists, masks, and toxic etchants are often not well‐suited for industrial production. Here, a cost‐effective stamping strategy is developed for scalable and rapid preparation of graphene‐based planar MSCs. Combining stamps with desired shapes and highly conductive graphene inks, flexible MSCs with controlled structures are prepared on arbitrary substrates without any metal current collectors, additives, and polymer binders. The interdigitated MSC exhibits high areal capacitance up to 21.7 mF cm^−2^ at a current of 0.5 mA and a high power density of 6 mW cm^−2^ at an energy density of 5 µWh cm^−2^. Moreover, the MSCs show outstanding cycling performance and remarkable flexibility over 10 000 charge–discharge cycles and 300 bending cycles. In addition, the capacitance and output voltage of the MSCs are easily adjustable through interconnection with well‐defined arrangements. The efficient, rapid manufacturing of the graphene‐based interdigital MSCs with outstanding flexibility, shape diversity, and high areal capacitance shows great potential in wearable and portable electronics.

## Graphical abstract

Highly flexible micro‐supercapacitors are manufactured on a large scale by a cost‐effective stamping strategy with highly conductive graphene inks. The efficient production of the flexible micro‐supercapacitor devices with outstanding shape diversity, high areal capacitance, and excellent cycling stability shows great potential for future applications in portable and wearable electronics.

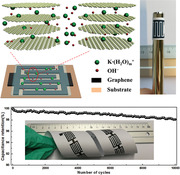

## 1 Introduction

Miniaturized electronics, especially flexible, smart, portable, and wearable microelectronics, are urgently demanding lightweight and thin energy storage devices at low cost with high voltage, high energy density, and outstanding flexibility.^[^ ^1^ ^]^ However, conventional micro‐supercapacitors (MSCs) and micro‐batteries with sandwich structures generally suffer from heavy weight, bulky volume, and limited flexibility. Moreover, the fixed shapes lead to inconvenient device connections and challenges in providing high capacitance and potential window requiring lots of conducting wires. Hence, conventional micro‐devices are hardly meeting the peculiar demands of high‐voltage electronics.^[^ ^2^ ^]^ Compared to conventional sandwich structures, planar MSCs with significant benefits such as long cycling life, fast rate capabilities, and superior power density have received tremendous interest as promising micro‐scale power sources for integrated electronic devices.^[^ ^3^ ^]^ More importantly, planar interdigital MSCs on‐chip with tailored sizes, designable shapes, superior flexibility, and space‐saving connections can potentially boost high‐voltage output.

To date, numerous efforts have been employed to explore novel electrode materials, including carbon‐based materials (such as carbon nanotubes,^[^ ^4^ ^]^ onion‐like carbon,^[^ ^5^ ^]^ activated carbon,^[^ ^6^ ^]^ and graphene^[^ ^7^ ^]^) and pseudocapacitive materials (such as NiO,^[^ ^8^ ^]^ MnO_2_,^[^ ^9^ ^]^ RuO_2_,^[^ ^10^ ^]^ poly(3,4‐ethylenedioxythiophene),^[^ ^11^ ^]^ polypyrrole,^[^ ^12^ ^]^ and polyaniline^[^ ^13^ ^]^), as thin‐film electrodes for supercapacitors (SCs). Among them, 2D materials (e.g., graphene, MXenes, phosphorene) have been studied and used as electrodes for MSCs due to their ultra‐thin thickness, large surface area, and mechanical robustness and flexibility, allowing for ultrafast shuttling of electrolyte ions along the plane of 2D nanosheets.^[^ ^14^ ^]^ Through rapid and reversible adsorption/desorption, ions are stored at the electrode/electrolyte interfaces without chemical reactions, resulting in superior cycling stability and ultrahigh power density (>10 000 W kg^−1^).^[^ ^15^ ^]^ In addition, their flexibility and nontoxic and nonhazardous features could fulfill stringent requirements of wearable and portable electronic devices. As a result, planar graphene MSCs based on electric double layer capacitance have shown great potential for the electronic industry, offering a fast charge–discharge rate, high power density, and long cycling life with a planar architecture compatible with integrated electronic circuits.

The fabrication of electrode‐patterned MSC devices often contains complex lithography processes (e.g., spin‐coating, masked irradiation, soft/hard baking, and development), high‐pressure pressing, O_2_ plasma etching, and current collector deposition (e.g., Cr, Au).^[^ ^16^ ^]^ Furthermore, the metal current collectors and interconnects lead to reduced volumetric and gravimetric capacitances, and ungratified flexibility of MSCs. A detailed comparison of the differences of the various techniques is shown in Table S1, Supporting Information. Therefore, several key challenges remain in the rational engineering of planar MSC devices, such as: 1) developing novel electrode materials (including current collectors) with outstanding energy and excellent flexibility; 2) exploring new electrolytes with broad voltage window for promoting energy density; and 3) simplifying the fabrication processes.

Here, we report a low‐cost and facile stamping approach to fabricate flexible planar MSCs using porous graphene inks. The resulting planar devices are free of metal current collectors, polymer binders, and separators. Notably, the stamped devices exhibited excellent uniformity, good electrical conductivity (170 S cm^−1^), remarkable mechanical flexibility, and strong adhesion with the cellulose paper. Both the manufacturing time and cost of the stamping strategy to fabricate graphene‐based MSCs are radically reduced compared to those of 3D printing and photolithography, which generally need certain requirements of the substrate or heavily rely on precision equipment. The graphene‐based MSCs display a high areal capacitance (C/A) of 21.7 mF cm^−2^, an outstanding cycling stability with 98.8% retention after 10 000 charge–discharge cycles, an energy density of 5 µWh cm^−2^ at a power density of 6 mW cm^−2^, and robust mechanical flexibility without any apparent degradation under various deformed states. It is worth noting that the stamping strategy is feasible for mass production of multiple flexible graphene‐based MSCs to regulate the capacitance and output voltage for integrated electronics.

## 2 Results and Discussion

**Figure 1:**
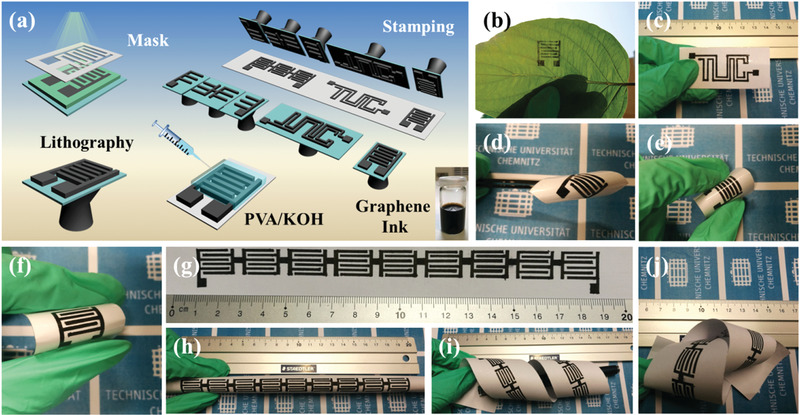
a) Schematic of the stamping fabrication of the flexible planar MSCs. b) MSCs stamped on a leaf. c) MSCs patterned with the text of “TUC.” d–f) Optical images of interdigital MSCs upon bending. g–j) Optical images of nine serially connected MSCs under flat (g), bending (h), spiral (i), and knotted (j) states.

The stamping strategy of graphene‐based MSCs is illustrated in **Figure** **1**. Stamps with desired shapes were produced onto clean silicon wafers by lithography with SU‐8 photoresist (Figure 1a). By using different designs of the shadow masks, the stamp shapes (e.g., interdigitated/TUC) and the finger numbers/widths/gaps, etc., could be precisely and reproducibly engineered. The stamps were treated by O_2_ plasma to obtain hydrophilic surfaces, so that the graphene ink could be easily brushed onto them. The graphene ink‐coated stamps were firmly pressed onto flexible substrates (such as cellulose paper and leaf, as shown in Figure 1b). After cast‐coating the gel electrolyte on the interdigital fingers, the MSC devices were dried at 25 °C for 12 h to form the all graphene‐based MSCs (Figure 1c). The as‐prepared micropatterns show robust mechanical flexibility under bending and twisting without structural degradation or delamination from the flexible substrates (Figure 1d–f). Notably, this flexible approach for fabricating MSCs is scalable and convenient for mass manufacturing of integrated MSCs (no additional current collectors, binders, and separators). Several graphene‐based MSCs connected in series were easily fabricated into a tandem cell pack (Figure 1g), demonstrating outstanding flexibility under various bending states, such as a spiral or a knotted shape (Figure 1h–j).

The schematic illustration of the fabrication of the highly porous and conductive graphene is shown in Figure S1, Supporting Information. A template‐assisted chemical vapor deposition (CVD) strategy was applied to synthesize porous graphene nanosheets. By utilizing a porous hexagonal MgO template, the porous graphene with good structural stability could be grown via CVD with several graphene layers, hexagonal morphologies, and large surface area. The fabrication strategy could be readily scaled up to achieve large‐scale preparation of the graphene with well‐defined structures. Because of its novel porous nanostructure and ultrahigh surface area, the porous graphene has an outstanding energy storage capability.

**Figure 2:**
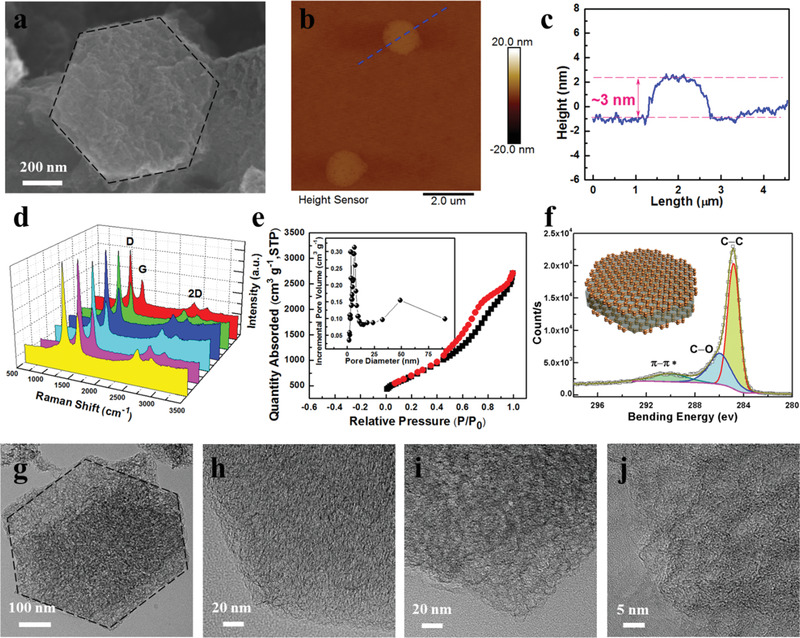
a) SEM images of porous graphene. b) AFM image and c) height profile of porous graphene flakes on a silicon wafer. d) Raman spectra of hexagonal porous graphene at different locations. e) Nitrogen adsorption–desorption isotherms and pore size distribution of porous graphene. f) C1s XPS spectrum of porous graphene. g–j) TEM images of hexagonal porous graphene.

The structure and morphology of as‐prepared graphene are characterized in **Figure** **2**. Scanning electron microscopy (SEM) images in Figure S4a–c, Supporting Information, show that the MgO@graphene samples consist of hexagonal flakes with an average size of ≈1 µm. After the template removal, the hexagonal structure is maintained. The flakes show highly porous and rough surfaces (Figure 2a; Figure S4d–f, Supporting Information). The thickness of the as‐prepared graphene flakes was studied by atomic force microscopy (AFM). The height profile of porous graphene indicates that the thickness of the flakes is about 3 nm (Figure 2c), corresponding to eight layers of graphene. Raman spectroscopy was performed to investigate the structural properties, homogeneity, and the quality of the as‐prepared porous graphene at different locations, as shown in Figure 2d. Typical Raman peaks of porous graphene are revealed in Figure 2d with a characteristic D peak (≈1340 cm), G peak (≈1580 cm), and 2D peak (≈2700 cm). The intensity ratio of the D and G bands (*I*_D_/*I*_G_) of the porous graphene is around 2, indicating a high density of defects of the template‐assisted CVD‐grown graphene. The powder X‐ray diffraction (XRD) patterns in Figure S2, Supporting Information, show crystalline structures of the MgO@graphene and porous graphene. The diffraction peaks observed at 36.9°, 42.9°, 62.3°, 74.6°, and 78.6° are assigned to the (111), (200), (220), (311), and (222) planes of MgO (JCPDS card no. 78‐0430). After acid etching, all the diffraction peaks of MgO disappeared (Figure S2b, Supporting Information); the graphene peak is dramatically broadened and of less intensity, indicating the presence of a high density of pores in the porous graphene sheets.

The nitrogen adsorption–desorption isotherms as well as the corresponding pore size distribution of the MgO@graphene and porous graphene are shown in Figure S3, Supporting Information, and Figure 2e. The porous graphene displays an ultrahigh Brunauer–Emmett–Teller (BET) surface area (2326 m^2^ g^−1^), much higher than that of MgO@graphene (140 m^2^ g^−1^). Furthermore, the distribution of pore sizes indicates a mesoporous feature with pore sizes in the range of 3−20 nm. The highly porous structure, high BET surface area, and wide pore size distribution can provide efficient transport paths as well as abundant active sites for electrons and ions, resulting in a better contact with electrolyte and therefore accelerating the ion transfer during the charge–discharge process. The graphene sample was further investigated by X‐ray photoelectron spectroscopy (XPS) that confirms the complete removal of the template. The XPS survey spectrum (Figure S6e, Supporting Information) exhibits predominantly the C 1s peak from graphene and no traces of metal species are detected. Figure 2f shows the C1s spectrum of the porous graphene with well‐fitted peaks, which are assigned to the C─C and/or the C═C (284.8 eV), C─O (hydroxyl and epoxide) group (286.0 eV), and the *π*–π* shake‐up satellite peak (291.0 eV), respectively. Transmission electron microscopy (TEM) images in Figure 2g–j and Figure S5, Supporting Information, show that the porous graphene possesses a hexagonal shape with high transparency. In addition, the enlarged TEM image confirms the porous structure, which is in good agreement with the SEM and N_2_ adsorption–desorption results.

For electronic applications, the porous graphene can be provided in dispersions or inks to prepare large‐area graphene conductive films on glass substrates. The porous graphene was pasted and pressed to form a flexible, continuous graphene film (≈50 µm in thickness) on transparent glass substrates (Figure S7b, Supporting Information). The van der Pauw measurement technique was performed to evaluate the electrical conductivity of the graphene film (Figure S7a, Supporting Information). The current, voltage, and the corresponding resistances of the graphene film are recorded in Table S2, Supporting Information, respectively. The calculated sheet resistance of the graphene film is around 1.18 Ω m^2^ (electrical conductivity: 170 S cm^−1^). The excellent electrical conductivity ensures rapid electron transport, resulting in high energy and power density of the porous graphene electrodes.

The electrochemical property of the porous graphene electrode was studied in a three‐electrode system in 6 M KOH electrolyte (Figure S8, Supporting Information). Figure S8a,b, Supporting Information, show the cyclic voltammogram (CV) curves tested at various scan rates from 25 to 1000 mV s^−1^ with a potential window of 0–0.8 V. It is clearly seen that the CV curves exhibit nearly rectangle shapes even at a high scan rate of 1000 mV s^−1^, and galvanostatic charge–discharge (GCD) curves show symmetric triangle shapes and negligible voltage drops, suggesting an ideal electric double layer capacitive feature. We further tested the electrical properties within a potential window of −0.6 to 0 V. The CV and GCD curves also show rectangle shapes and symmetric triangle shapes. The electrode displays a specific capacitance of 130 F g^−1^ at 1 mA with both positive and negative potential windows, suggesting outstanding capacitive properties, remarkable reversibility, and a broad operation potential window. The long‐term cycling stability of the porous graphene electrode was further investigated by GCD tests at 5 mA within a potential window of 0–0.8 V and −0.6 to 0 V. The cycling curves in Figure S9, Supporting Information, demonstrate that 98.5% and 98.9% retention of the capacitance are maintained after 5000 cycles within these two potential windows, showing outstanding cycling stability. The shapes of the last 10 GCD curves are maintained well after 5000 cycles (insets in Figure S9a,b, Supporting Information). In addition, the very small voltage drops further demonstrate the excellent electrochemical reversibility.

**Figure 3:**
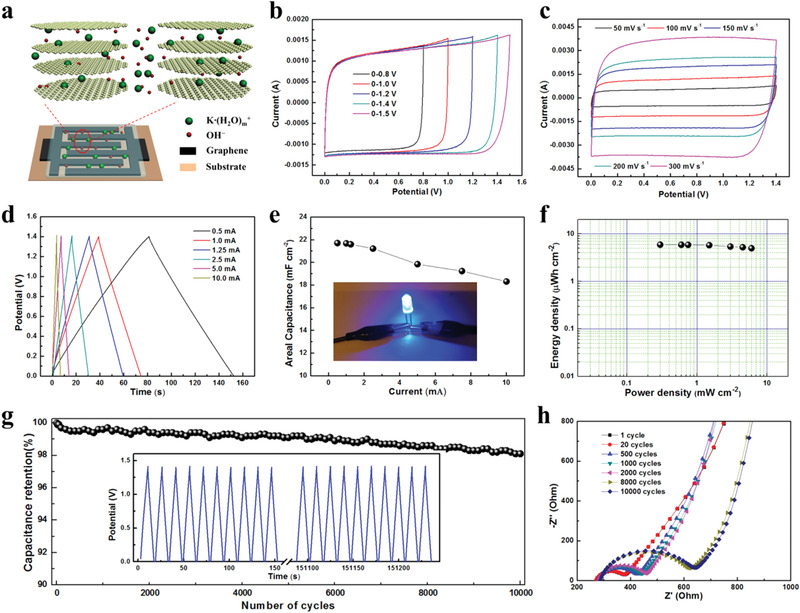
Electrochemical properties of the interdigital MSCs. a) Illustration of charge storage mechanism of the graphene‐based MSC. b) CV curves of MSCs tested at various potential windows. c) CV curves of MSCs tested at different scan rates. d) GCD curves of MSCs measured at various current densities. e) Areal capacitance at different current densities. Inset shows the photograph of the MSCs in series lighting a LED. f) Ragone plot of graphene‐based MSC. g) Cycling performance of the MSCs. Inset shows the first and final 10 GCD curves of the 10 000 cycles. h) Electrochemical impedance spectra of the MSCs after different charge–discharge cycles.

The porous graphene ink was stamped onto a piece of cellulose paper to form symmetric MSCs in which PVA/KOH gel electrolyte was employed to seal the patterned electrodes (**Figure** **3**a). The surface area of the interdigital electrodes is about 1.17 cm^2^ and the thickness is around 20 µm. The electrode size parameters of the interdigital MSC are shown in Figure S11, Supporting Information. Figure 3b shows the CV curves of the graphene‐based MSC measured at 100 mV s^−1^ within different potential windows. A maximum potential window of 0–1.5 V was employed, beyond which the CV curves show distorted features, indicating irreversible reactions on the electrode surface. Hence, a potential voltage of 0–1.4 V was used as the safe and optimal operating voltage for the MSCs. The electrochemical performance of the stamping MSCs was evaluated by CV and GCD measurements in Figure 3c,d. It is notable that CV curves (Figure 3c; Figure S12, Supporting Information) retain a nearly rectangular shape at scan rates ranging from 50 to 1600 mV s^−1^, and display a linear dependence of the maximum current density with scan rates, manifesting high electrochemical reversibility and high‐power capability. The absence of redox peaks indicates a double‐layer capacitive behavior of the graphene‐based electrodes. The GCD curves of the graphene‐based interdigital MSC (Figure 3d) exhibit a symmetric shape with a small voltage drop. The interdigital MSCs show outstanding areal capacitances of 22.5 mF cm^−2^ at 300 mV s^−1^ and 21.7 mF cm^−2^ at 0.43 mA cm^−2^. When the current density is increased to 8.5 mA cm^−2^, the areal capacitance stays at 84.3% (18.3 mF cm^−2^) of the original value measured at 0.43 mA cm^−2^. The volumetric capacitance is calculated to be 10.9 F cm^−3^ at a current density of 0.22 A cm^−3^ for the interdigital electrode. The rate capability of the graphene‐based interdigital MSC is shown in Figure 3e. The remarkable rate capability is attributed to the firm electrical contacts between the current substrate and the graphene electrodes, good conductivity (170 S cm^−1^) and large surface area (2326 m^2^ g^−1^) of the active materials.

Energy densities and power densities are essential parameters to evaluate the performance of energy storage devices. The Ragone plot in Figure 3f displays the areal energy and power densities of the graphene‐based MSCs. The maximum areal energy of the MSC device is 5.9 µWh cm^−2^ at a power density of 0.3 mW cm^−2^, corresponding to a volumetric energy density of 2.95 mWh cm^−3^ at a volumetric power density of 149 mW cm^−3^. The maximum areal power density is 6.0 mW cm^−2^ at an energy density of 5.0 µWh cm^−2^, corresponding to a volumetric power density of 3.0 W cm^−3^ at a volumetric energy density of 2.5 mWh cm^−3^. Table S4, Supporting Information, shows the detailed comparison of the voltage window, capacitance, cycling life, energy, and power density of recently reported graphene‐based MSCs, among which our device shows superior electrochemical performance compared to most of these works.^[^ ^17^ ^]^

The long‐term cycling property of our device was evaluated by GCD tests at 5 mA within an operating voltage from 0 to 1.4 V (Figure 3g). The areal capacitance maintains 98.8% after 10 000 cycles, demonstrating an outstanding cycling stability. Notably, the last 10 GCD curves maintain their shapes well after long‐term cycling (inset in Figure 3g). XPS was employed to evaluate the wetting and penetration of electrolyte ions (K^+^) into the electrode materials during the electrochemical tests (Figure S13, Supporting Information). The K2p spectra were collected during XPS depth profiling (etching depth was up to several hundreds of nanometers) from both sides of the electrode (Figure S13c, Supporting Information), clearly revealing the homogeneous penetration of K^+^ through the whole electrode. The calculated atomic ratio of K/C does not show any significant change versus depth, further indicating the good contact with the electrolyte and efficient transport paths of the electrolyte ions in the electrode materials during the electrochemical process. Meanwhile, the SEM image of the porous graphene after 10 000 cycles (Figure S14, Supporting Information) suggests that the porous structure is maintained very well.

The electrochemical resistance was further evaluated by electrochemical impedance spectroscopy (EIS) measured after different charge–discharge cycles. In the high‐frequency regions of the Nyquist plot in Figure 3h, the rising diameter of the semicircles indicates the slow increase of the charge transfer resistance (Rct) from 77.3 to 209.1 Ω after 10 000 cycles. Furthermore, internal resistance (Rs) can be calculated by the intercept of the curves with the *x*‐axis, which shows a slight increase from 264.2 to 281.6 Ω after 10 000 cycles, suggesting superior conductivity of the electrolyte and the small internal resistance of the electrodes. More detailed results of the Rct and Rs of the graphene‐based MSC after cycling are recorded in Table S3, Supporting Information.

**Figure 4:**
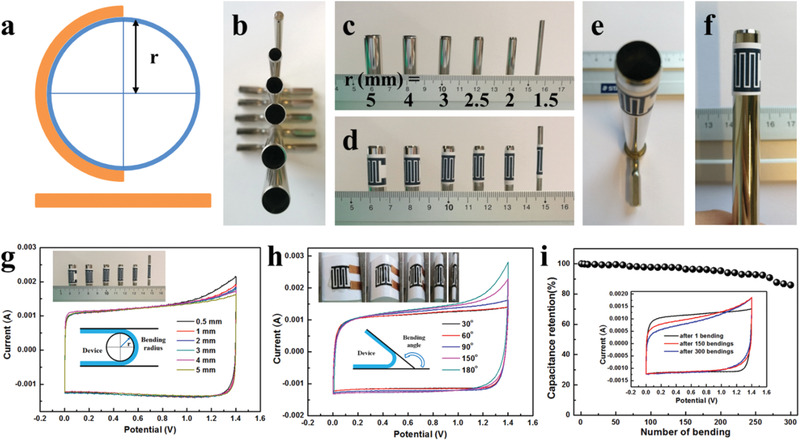
Electrochemical stability of graphene‐based MSCs during bending tests. a) Schematic illustration of the bending test. b,c) Photographs of bending supports with different bending radii. d–f) Representative photographs of MSCs bending at bending radii from 1.5 to 5 mm. g,h) CV curves tested at a scan rate of 100 mV s^−1^ for different bending radii (g) and different bending angles (h). i) Capacitance retention as a function of bending cycles.

The mechanical properties of the graphene‐based MSCs were characterized by bending tests. Both bending angle and bending radius (*r*) (or bending curvature 1/*r*) are used to characterize the mechanical properties (**Figure** **4**a–f). To demonstrate the remarkable flexibility of the MSC, we examined the CV curves of graphene‐based MSCs under different bending states (radius from 1.5 to 5 mm) (Figure 4g). All the CV curves of the graphene‐based MSCs overlap well, demonstrating excellent flexibility and electrochemical stability. We also compared the electrochemical performance of the graphene‐based MSC at different bending states with bending angles in the range of 0°–180° (Figure 4h). It is notable that all the CV curves remain nearly unchanged and show negligible capacitance fluctuations even when the device was strongly bent. The weak polarization may be due to a minor damage of the electrode material/substrate and electrolyte flow during the bending process. The cycling stability of the graphene‐based MSC was measured at a current of 5 mA after 300 bending cycles (with a bending angle of 180°) (Figures 4i). The areal capacitance of the graphene‐based MSC maintains ≈83.5% of the original capacitance after 300 cycles. Such remarkable flexibility is mainly caused by the special design, the highly porous structure, and large surface area of graphene flakes and the outstanding integrity of the microelectrodes and interconnects, offering enormous potential to seamless integration of the graphene‐based MSC with flexible microelectronics.

**Figure 5:**
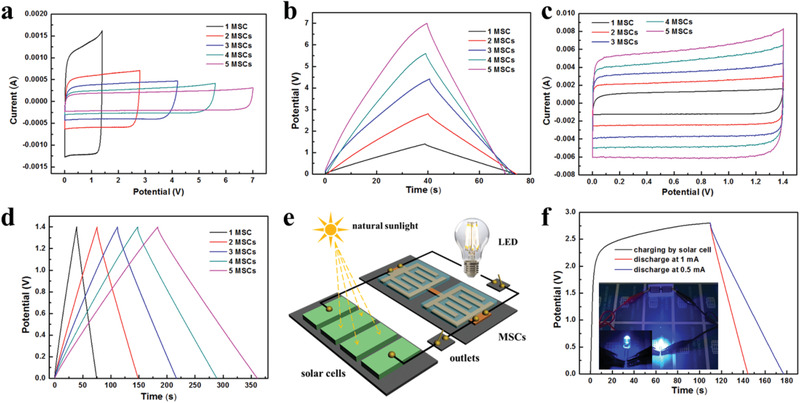
a) CV curves at a scan rate of 100 mV s^−1^ and b) GCD curves at a current of 1 mA of integrated graphene‐based MSCs with different numbers of devices connected in series. c) CV curves at a scan rate of 100 mV s^−1^ and d) GCD curves at 1 mA of different integrated graphene‐based MSCs connected in parallel. e) Schematic of the MSCs bridging solar cells for solar energy storage. f) Charging curve (black) of the MSCs charged by commercial solar cells, and discharging curves (blue and red) of the MSCs at different current. Inset shows a LED lightened by the energy stored in MSCs.

To satisfy the increasing demands of integrated electronics, microdevice packs with high energy and tailored voltage are produced via the integration of multiple graphene‐based MSCs connected in parallel and in series. We have to emphasize that the strategy of one‐step stamping manufacturing as well as continuous integration of the device arrays is efficient and highly scalable for large‐scale production without involving extra steps. As expected, both CV curves (**Figure** **5**a) and GCD profiles (Figure 5b) show a stepwise increase of the operating voltages from 1.4 V (one single cell) to 7.0 V (five cells connected in series), indicating an exceptional performance uniformity. In addition, the maximum currents of the CV profiles (Figure 5c) and charge–discharge time of the GCD curves (Figure 5d) of the graphene‐based MSCs in parallel (from one to five cells) increase linearly. The rapid manufacturing of our integrated devices demonstrates enormous potential for microelectronics industry. Remarkably, two serially connected micro‐devices can light a blue light‐emitting diode (LED) (rated voltage of 1.9 V) as standalone power sources (Figure S15a,b, Supporting Information). After being charged for 7 s, the connected devices can power the LED for about 35 s. Hence, ultra‐compact MSCs modules with tunable output voltages and capacitance can be readily obtained by serial and parallel connections.

In order to further evaluate the potential use of the MSCs in on‐chip electronics, serially connected micro‐devices can be readily integrated with solar cells to build a self‐powered configuration to store solar energy. Our flexible devices were employed as a bridge to connect a blue LED with two serially connected commercial Si‐based solar cells (open‐circuit potential (OCP): 1.5 V). Figure 5e and Figure S15c, Supporting Information, show the schematic and photograph of the detailed electric circuits. The commercial solar cells (power source) were first used to charge the flexible devices under sunlight for about 118 s to achieve an OCP of 2.8 V (Figure 5f). Afterwards, an electrochemical workstation was employed to evaluate the discharge behavior of the charged MSCs at different current of 0.5 and 1 mA. Figure 5f (inset) and Figure S15d, Supporting Information, show that the LED was successfully lightened by the energy stored in the serially connected MSCs.

## 3 Conclusion

In summary, a highly flexible graphene‐based MSC was manufactured by a cost‐effective stamping strategy without metal current collectors, binders, or additives. The interdigitated MSC exhibits remarkable electrochemical performance owing to the rational design of the device, high electrical conductivity (170 S cm^−1^), and large surface area (2326 m^2^ g^−1^) of the active materials. A high areal capacitance up to 21.7 mF cm^−2^ was obtained at a current of 0.5 mA. Notably, the devices exhibit remarkable cycling stability and remarkable flexibility over 10 000 charge–discharge cycles and 300 bending cycles (with a bending radius of 1 cm and bending angle of 180°). In addition, the output voltage and capacitance of the MSCs are easily adjustable through interconnection with well‐defined arrangements. Therefore, the efficient production of the flexible graphene‐based MSCs with outstanding flexibility, shape diversity, and high areal capacitance shows great potential for future applications in portable and wearable electronics.

## 4 Experimental Section

##### Synthesis of MgO@Graphene

The magnesium hydrate (Mg(OH)_2_, as purchased from Sigma‐Aldrich) powder was loaded in a alumina boat, which was then transferred to a clean quartz tube in the CVD system. The system was first pumped down to a base pressure of around 0.2 mTorr, followed by two purges in pure Ar, and then 50 sccm of Ar was introduced to the system to achieve a pressure of 200 Torr. The CVD system was gradually heated to 1030 °C for 1 h and maintained for another hour and then the Ar flow was switched off and a mixed flow of CH_4_ (50 sccm) and H_2_ (30 sccm) was introduced into the system for the graphene growth for 1.5 h. After growth, CH_4_ and H_2_ gas were switched off and Ar was switched on and the system was quickly cooled down to a room temperature.

##### Synthesis of Porous Graphene

The as‐obtained MgO@graphene sample was dispersed in aqueous HCl solution (3 m) and followed by a constant stirring at a hot plate of 80 °C for 24 h to remove the MgO template. The porous graphene was collected by filtration, washed with deionized water several times, and freeze‐dried.

##### Fabrication of the Stamps

The stamps were patterned onto the clean silicon wafer with lift‐off protocol SU‐8 photoresist by photolithography. SU‐8 frame was first built between and around the interdigital gold current collectors, and in the same manner, SU‐8 3000 resist was spin coated on the substrate at 500 rpm for 8 s. After baking at 90 °C for 20 min and 120 °C for 40 min, the sample was exposed with a designed glass/Cr photomask by using SUSS MJB4 mask aligner. Thereafter, the sample was baked at 95 °C for another 30 min. The developing process was performed in propylene glycol methyl ether acetate (micro resist technology GmbH) to remove unexposed polymer. Finally, the SU‐8 stamps were treated by oxygen plasma for 10 min prior to the stamping process to increase the hydrophilicity. The thickness of the SU‐8 frame was around 1 mm.

##### Stamping of the Graphene MSCs

Typically, the graphene ink was brushed onto the interdigitated fingers of stamp. Then the stamp was firmly pressed onto a piece of cellulose paper. After 3 s, the stamp was removed and the MSC was dried naturally. The stamping process was repeated three times. Other MSCs, such as “TUC” pattern, were stamped by stamping the graphene ink in a similar way. In addition, graphene ink was also stamped onto a leaf. All the MSCs were vacuum‐dried overnight before tests.

##### Materials Characterization

The crystallographic information and chemical composition of the samples were characterized by XRD (D/max 2500, Cu Ka) and XPS (Thermo Fisher Scientific, ESCALAB 250Xi). The morphology and microstructure of the products were investigated by SEM (ZEISS Auriga) and TEM (ZEISS LIBRA 200). Nitrogen adsorption–desorption isotherms were measured at 77 K with a Micromeritics ASAP 2020 sorptometer. Raman analysis was performed via a Raman spectrometer (LabRAM HR Evolution, HORIBA Scientific) at room temperature.

##### Electrochemical Measurements

The electrochemical tests including CV, GCD, and EIS of the MSCs were measured using an electrochemical workstation (autolabIII/FRA2) in 6 m PVA/KOH, 1 m PVA/H_2_SO_4_, and 1 m PVA/Na_2_SO_4_ gel electrolyte.

The areal/volumetric specific capacitances (*C*) of the samples can be calculated from both the GCD and CV curves based on the following equations

(1) $$C=\frac{I\times \Delta t}{A\times \Delta V}$$

(2) $$C=\frac{1}{A\times \mu \times \left(V_{2}-V_{1}\right)}\int_{V_{1}}^{V_{2}}IdV$$

where *A*, *I*, *∆t*, *∆V*, *µ*, *V*_1_, and *V*_2_ are the footprint areal (cm^2^)/volume (cm^3^) of the electrodes, discharge current (mA), the discharging time (*s*), the discharging potential range (*V*), the scan rate, high‐voltage value, and low‐voltage value, respectively.

Energy density (*E*, Wh cm^−2^/Wh cm^−3^) and power density (*P*, W cm^−2^/W cm^−3^) of the devices were calculated using the following equations

(3) $$E=\frac{C\times \Delta V^{2}}{7200}$$

(4) $$P=\frac{E}{\Delta t}\times 3600$$

## Conflict of Interest

The authors declare no conflict of interest.

## Supporting information

Supporting Information
