# Supplementary material for: Stamping Fabrication of Flexible Planar Micro‐Supercapacitors Using Porous Graphene Inks
Source: Adv Sci (Weinh). 2020 Jul 27;7(19):2001561. doi: 10.1002/advs.202001561 (PMC7539196; doi:10.1002/advs.202001561)
Supplement: Supplementary file 1 — Supporting Information [file ADVS-7-2001561-s001.pdf]

Copyright WILEY-VCH Verlag GmbH & Co. KGaA, 69469 Weinheim, Germany, 2020.

ADVANCED SCIENCE
Open Access

Supporting Information

for *Adv. Sci.*, DOI: 10.1002/advs.202001561

Stamping Fabrication of Flexible Planar Micro-Supercapacitors Using Porous Graphene Inks

Fei Li, Jiang Qu, Yang Li, Jinhui Wang, Minshen Zhu, Lixiang Liu, Jin Ge, Shengkai Duan, Tianming Li, Vineeth Kumar Bandari, Ming Huang, Feng Zhu,* and Oliver G. Schmidt**

Supporting Information

Stamping Fabrication of Flexible Planar Micro-Supercapacitors Using Porous Graphene Inks

Fei Li, Jiang Qu, Yang Li, Jinhui Wang, Minshen Zhu, Lixiang Liu, Jin Ge, Shengkai Duan, Tianming Li, Vineeth Kumar Bandari, Ming Huang, Feng Zhu*, Oliver G. Schmidt**

F. Li, J. Qu, Y. Li, J. Wang, L. Liu, S. Duan, T. Li, V. K. Bandari, Dr. F. Zhu, Prof. O. G. Schmidt

Material Systems for Nanoelectronics

Chemnitz University of Technology

09107 Chemnitz, Germany

E-mail: zhufeng@ciac.ac.cn; o.schmidt@ifw-dresden.de

Dr. M. Huang

School of Materials Science and Engineering

Ulsan National Institute of Science and Technology (UNIST)

Ulsan 44919, Republic of Korea

E-mail: xiaoming.huang694@gmail.com

Prof. F. Zhu

State Key Laboratory of Polymer Physics and Chemistry

Changchun Institute of Applied Chemistry, Chinese Academy of Sciences

130022 Changchun, P.R. China

F. Li, J. Qu, Y. Li, J. Wang, L. Liu, S. Duan, T. Li, V. K. Bandari, Dr. F. Zhu, Prof. O. G. Schmidt

Center for Materials, Architectures and Integration of Nanomembranes (MAIN),

Chemnitz University of Technology

09126 Chemnitz, Germany

F. Li, J. Qu, Y. Li, J. Wang, Dr. M. Zhu, L. Liu, Dr. J. Ge, S. Duan, T. Li, V. K. Bandari, Dr. F. Zhu, Prof. O. G. Schmidt

Institute for Integrative Nanosciences

Leibniz IFW Dresden

01069 Dresden, Germany

Prof. O. G. Schmidt

School of Science, Dresden University of Technology,

01062 Dresden, Germany

Table S1. Key features of various techniques for fabrication of MSCs.

MSCs	Cost	Throughput	Prerequisite(s)	Resolution	Materials
Stamping	Low	hHgh	Ink, stamps	200 µm	Polymer, paper
Ink-jet printing	Low	High	Ink viscosity	20 µm	Metal, ceramic, and polymer suspension
Screen printing	Low	High	Ink, plates	10 µm	Glass, ceramic
3D printing	High	High	-	-	Plastic, certain metals or ceramics
Laser writing	High	High	High power laser	200 µm	Polymer, metal, ceramic powders
Photolithography	High	Low	Digital dynamic masks	sub-one micron	UV-curable polymers
Electron beam lithography	High	Low	Electron beam	sub-one micron	Polymer, metal, ceramic
FIB etching	High	Low	Focused-ion-beam	sub-one micron	Metal, polymer

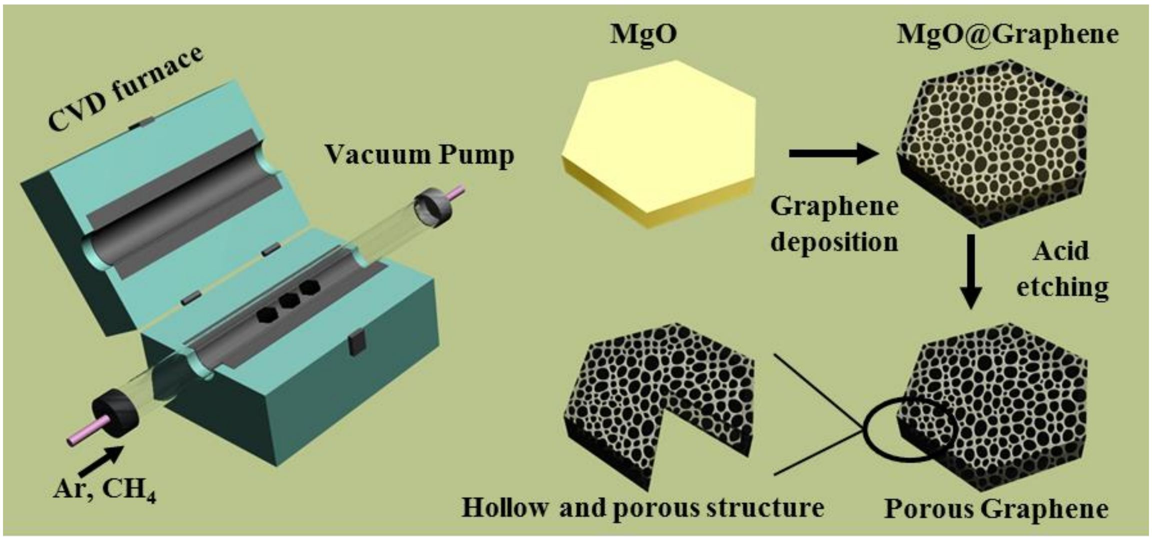

Figure S1. Illustration of the preparation of the polygonal porous graphene.

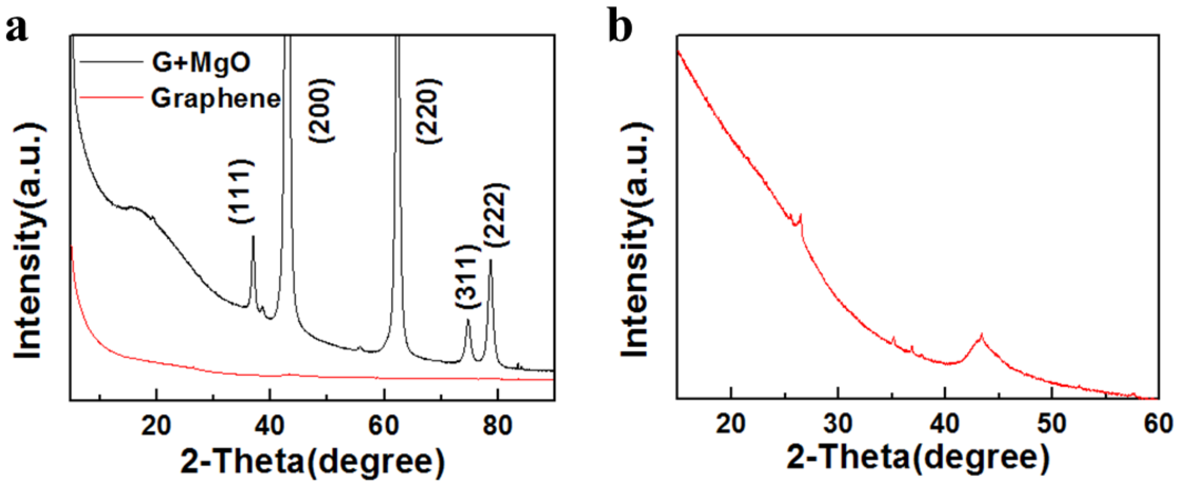

Figure S2. XRD patterns of MgO@graphene and porous graphene (a) and enlarged view of XRD pattern of porous graphene (b).

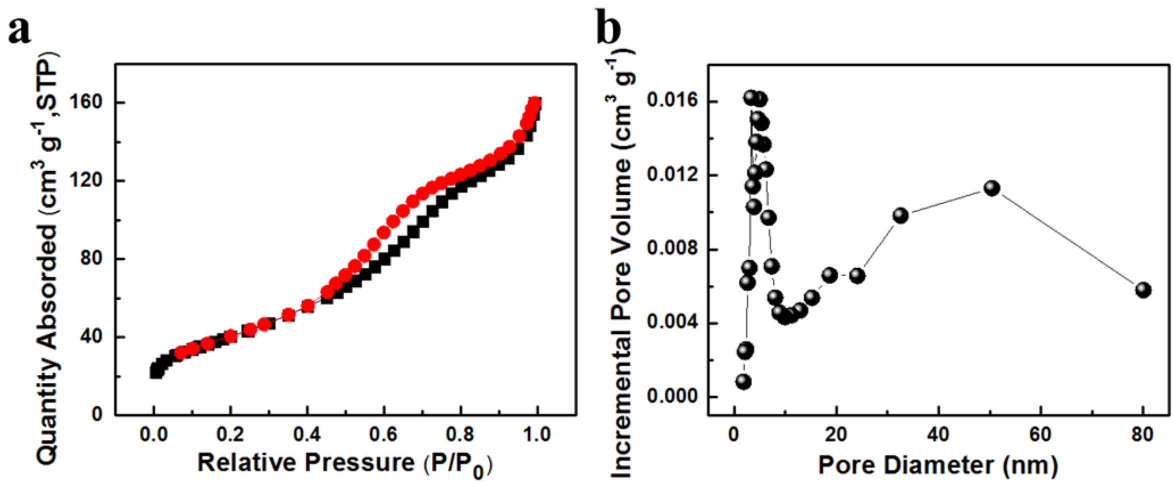

Figure S3. Nitrogen adsorption–desorption isotherms (a) and pore size distributions (b) of MgO@graphene.

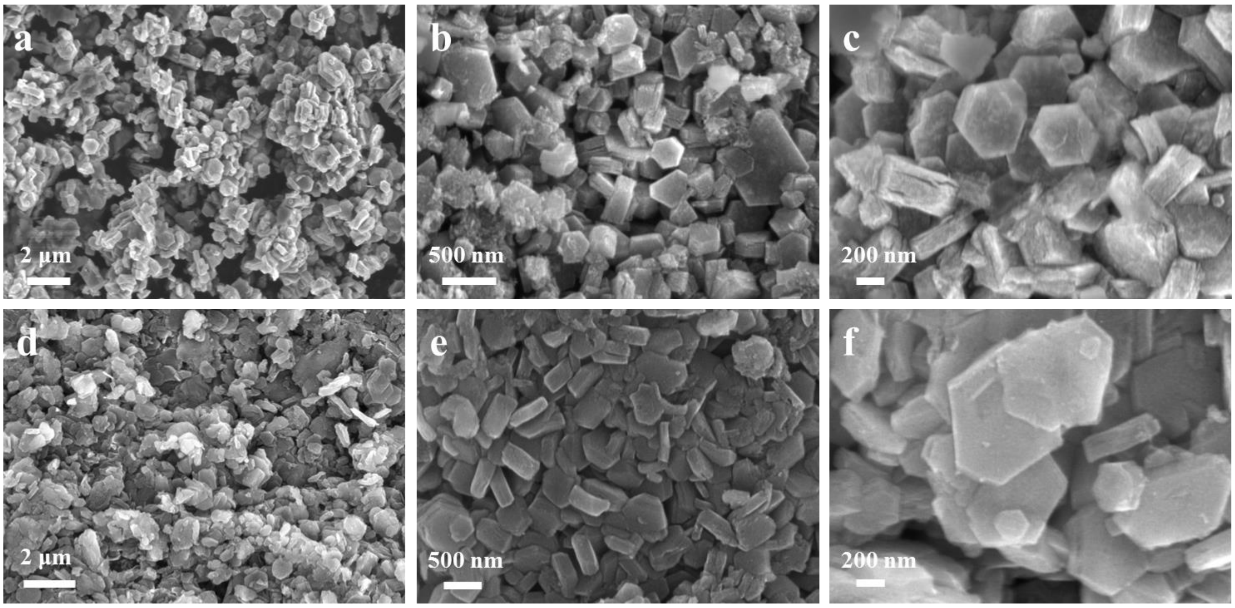

Figure S4. (a-c) SEM images of MgO@graphene; (d-f) SEM images of porous graphene after the removal of MgO template.

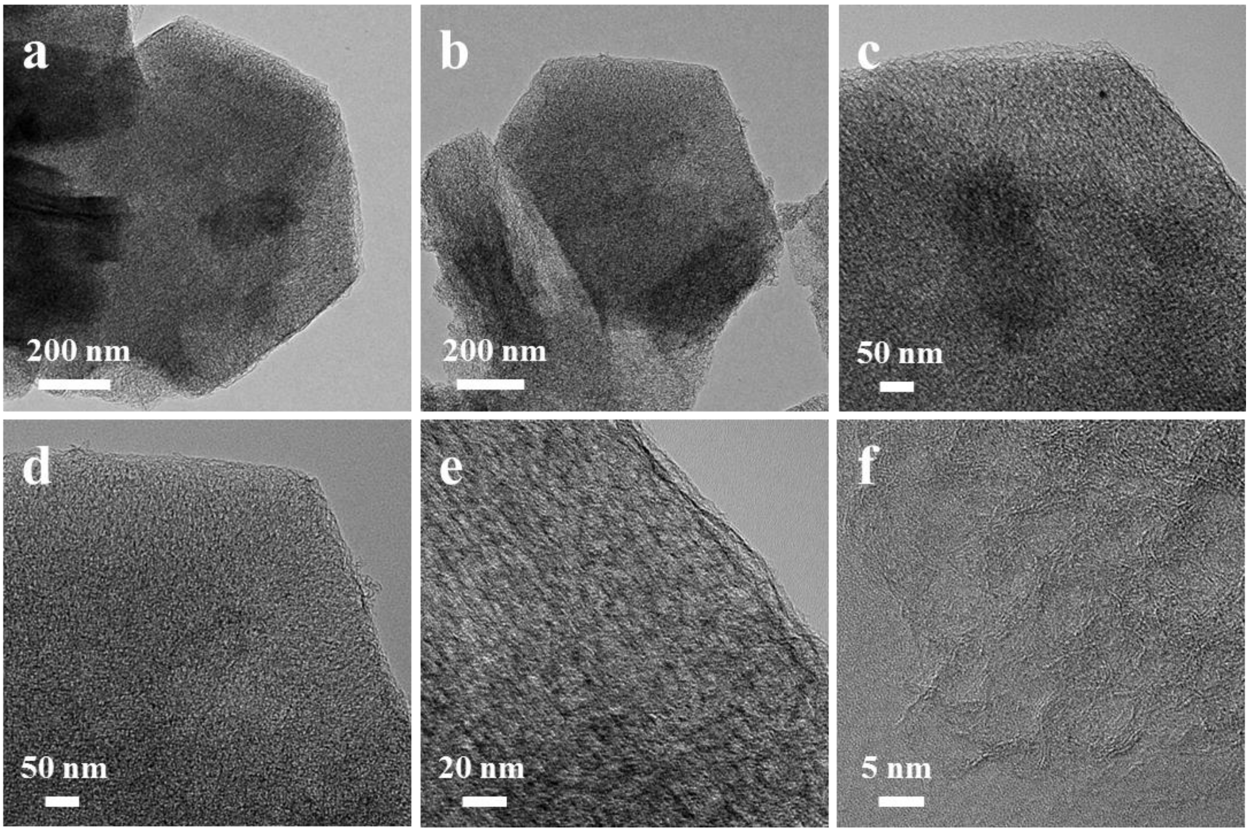

Figure S5. TEM images of (a-b) complete hexagonal porous graphene, (c-d) one corner and (e) one side of the hexagon hexagonal porous graphene; (f) HRTEM of the hexagonal porous graphene.

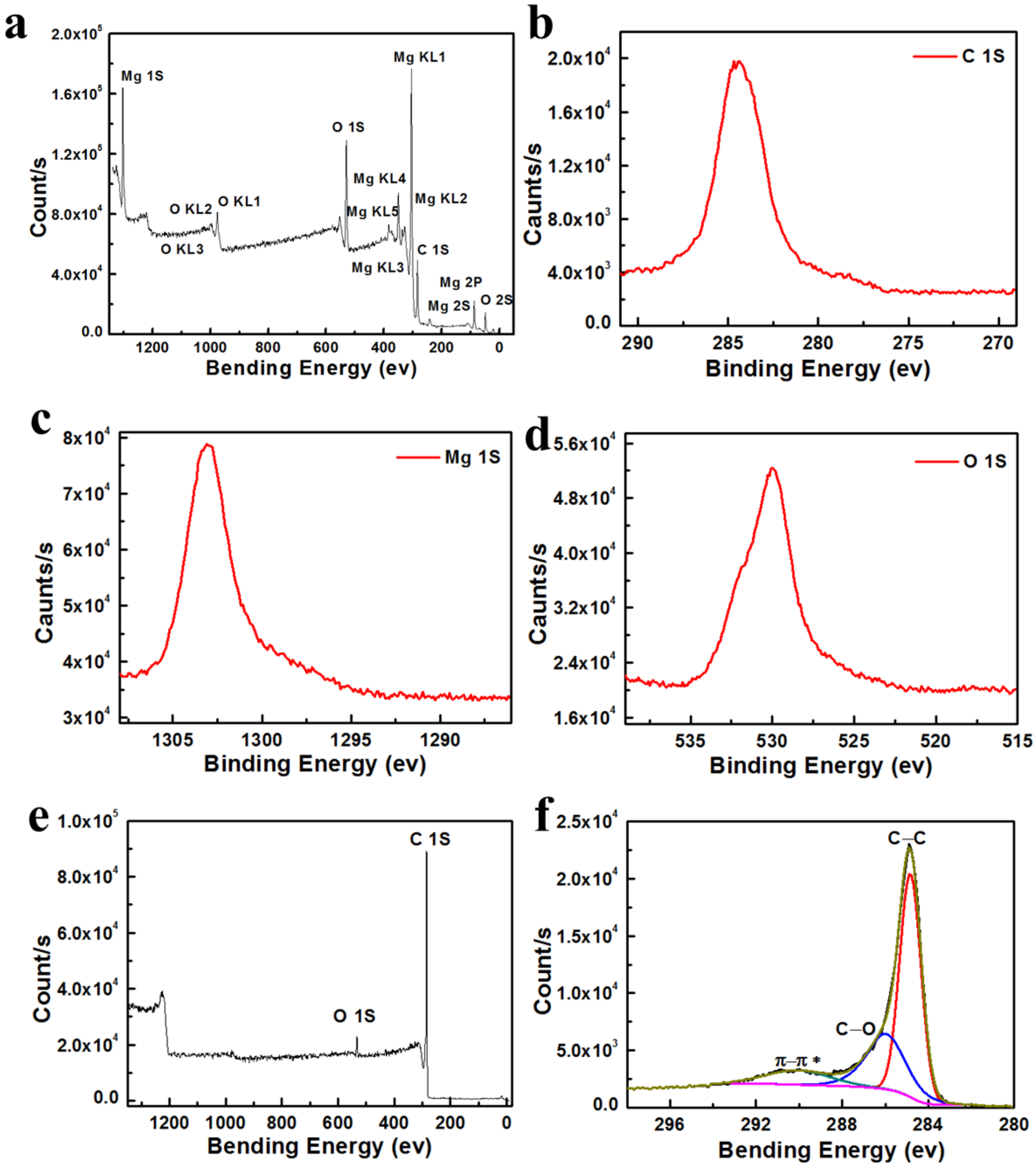

Figure S6. (a-d) XPS spectra of MgO@graphene; (e, f) XPS spectra of porous graphene.

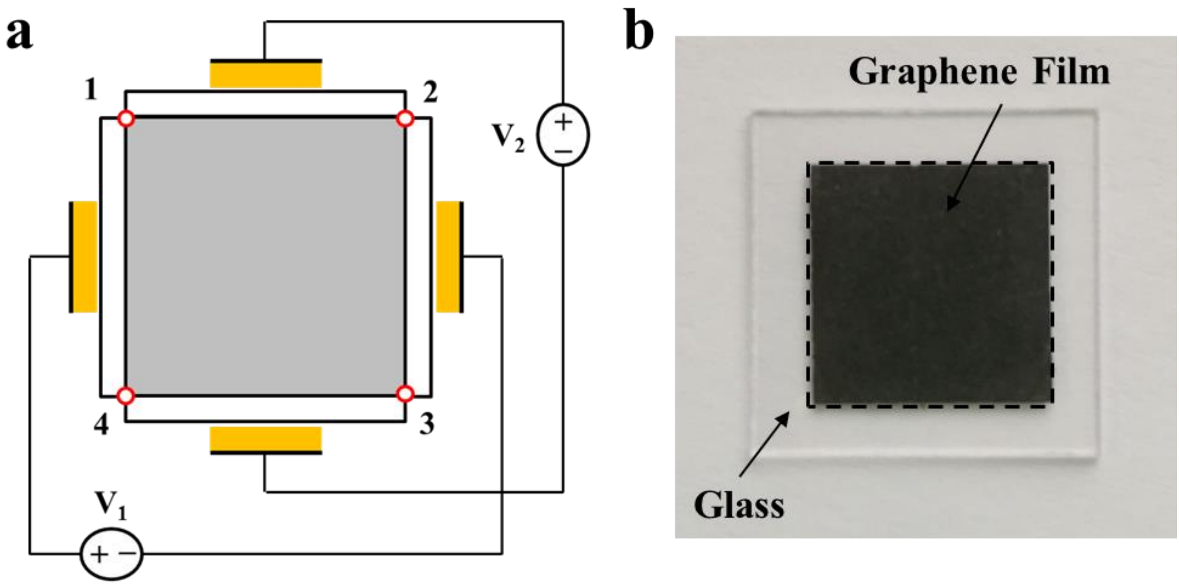

Figure S7. (a) Illustration of van der Pauw technique for the conductivity measurement of graphene film; (b) Photograph of a graphene film on glass.

Table S2. Current, voltage and resistance of the graphene film during the conductivity test.

	I		V		R
I_{12}	1 mA	V_{34}	0.248 mV	$R_{12,34}$	0.248 Ω
I_{34}	1 mA	V_{12}	0.270 mV	$R_{34,12}$	0.270 Ω
I_{23}	1 mA	V_{41}	0.266 mV	$R_{23,41}$	0.266 Ω
I_{41}	1 mA	V_{23}	0.254 mV	$R_{41,23}$	0.254 Ω

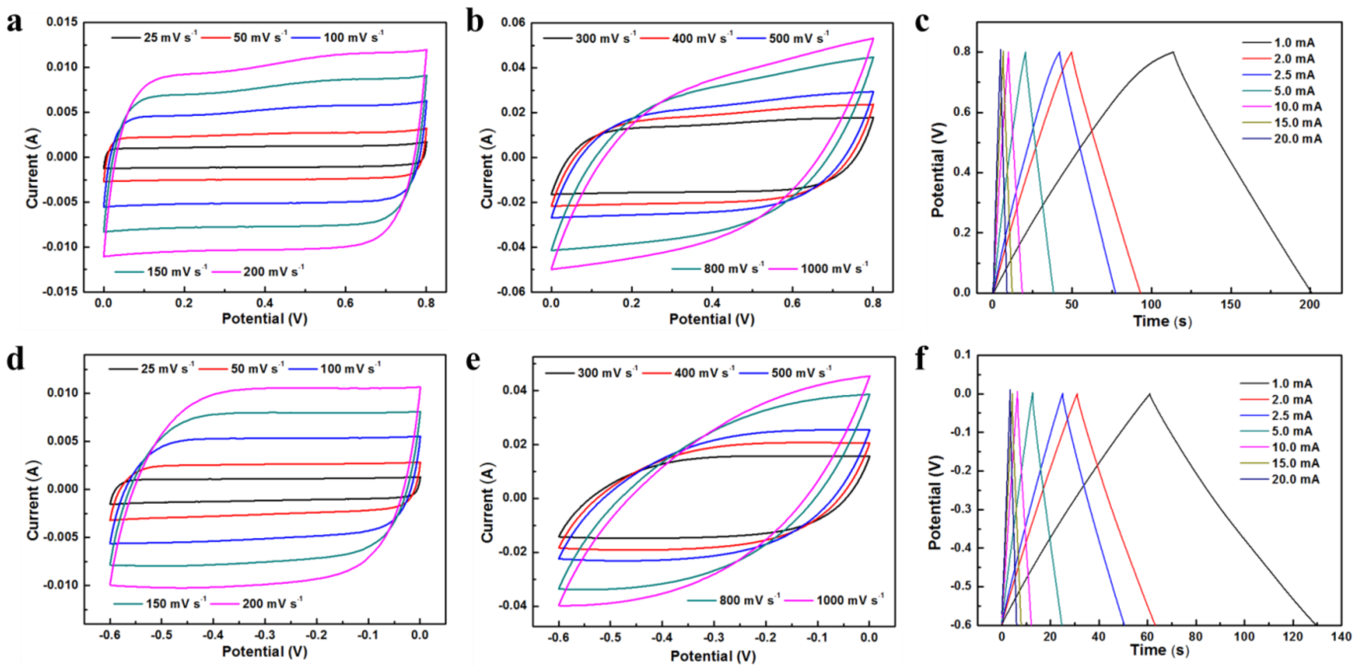

Figure S8. Electrochemical performance of the porous graphene measured in 6 M KOH electrolyte in three-electrode system. (a, b) CV curves of the graphene electrode at various scan rates with a potential window of 0-0.8V; (c) GCD curves of the MSC at different current densities; (c, d) CV curves of the graphene electrode at various scan rates with a potential window of −0.6-0V; (f) GCD curves of the MSC at different current densities with a potential window of −0.6-0V.

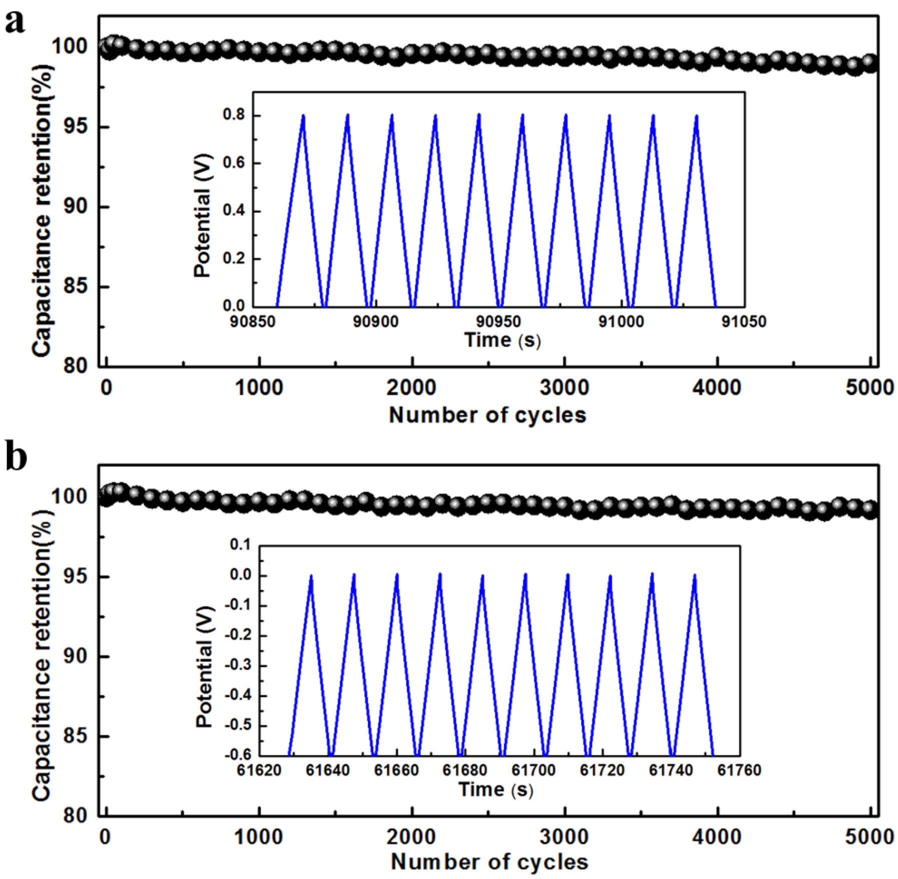

Figure S9. Cycling performance of the graphene electrodes at a current of 10 mA with the potential window of 0-0.8V and −0.6-0V in 6 M KOH electrolyte. Inset shows the final 10 GCD curves of the 5000 cycles.

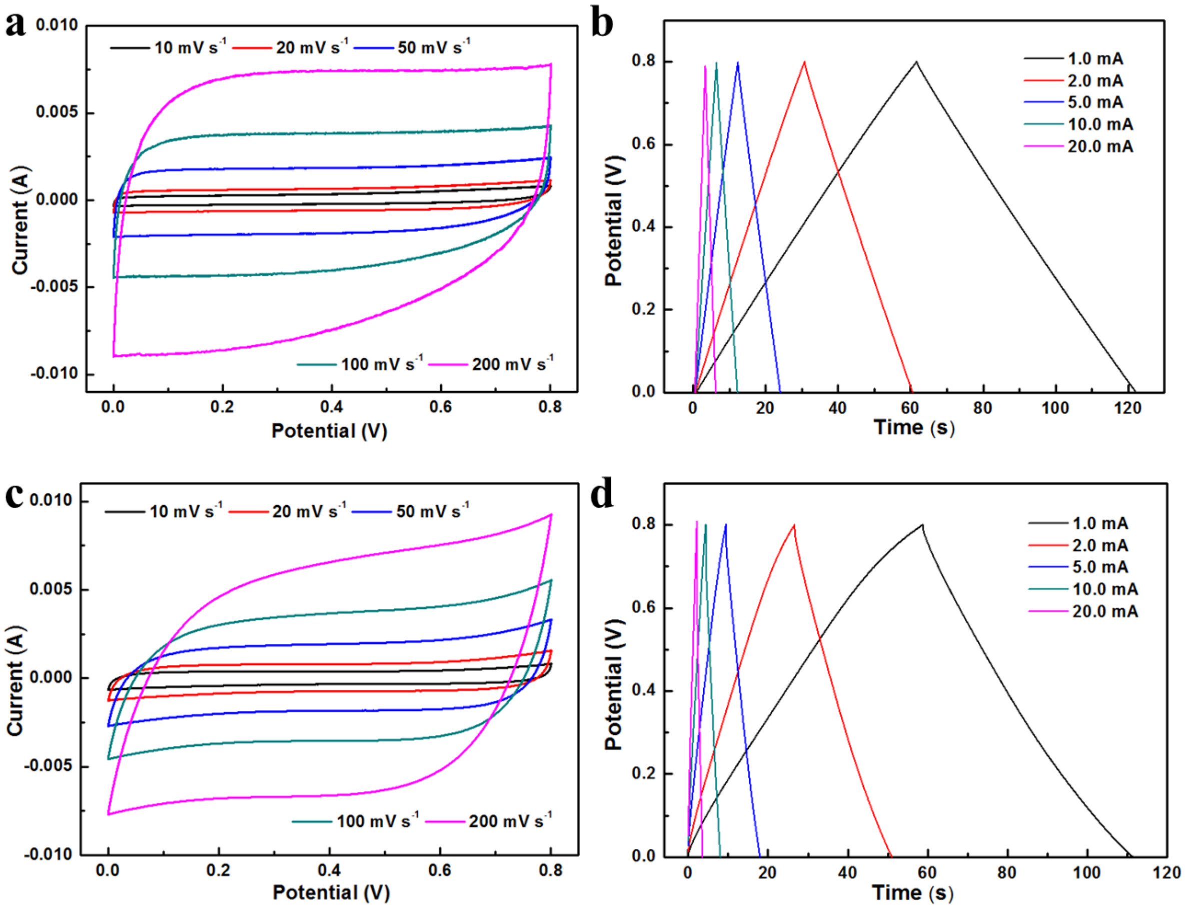

Figure S10. (a) CV curves at various scan rates and (b) GCD curves at different current of porous graphene measured in 1 M Na_2SO_4 electrolyte; (c) CV curves at various scan rates and (d) GCD curves at different current of porous graphene measured in 1 M H_2SO_4 electrolyte.

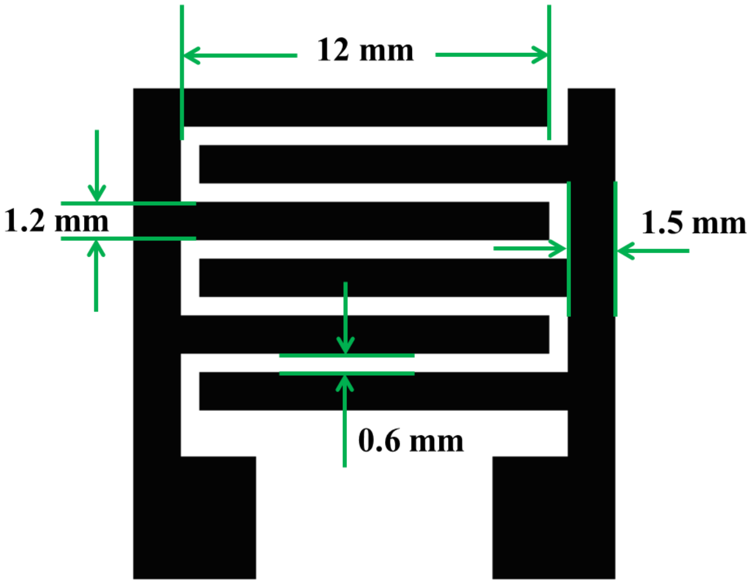

Figure S11. Microelectrode size parameters of interdigital MSCs.

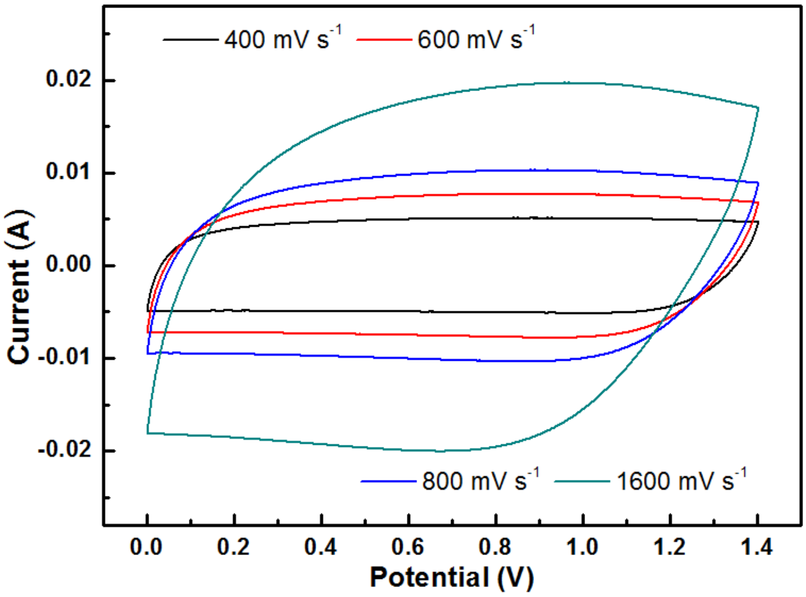

Figure S12. CV curves of MSCs tested at high scan rates.

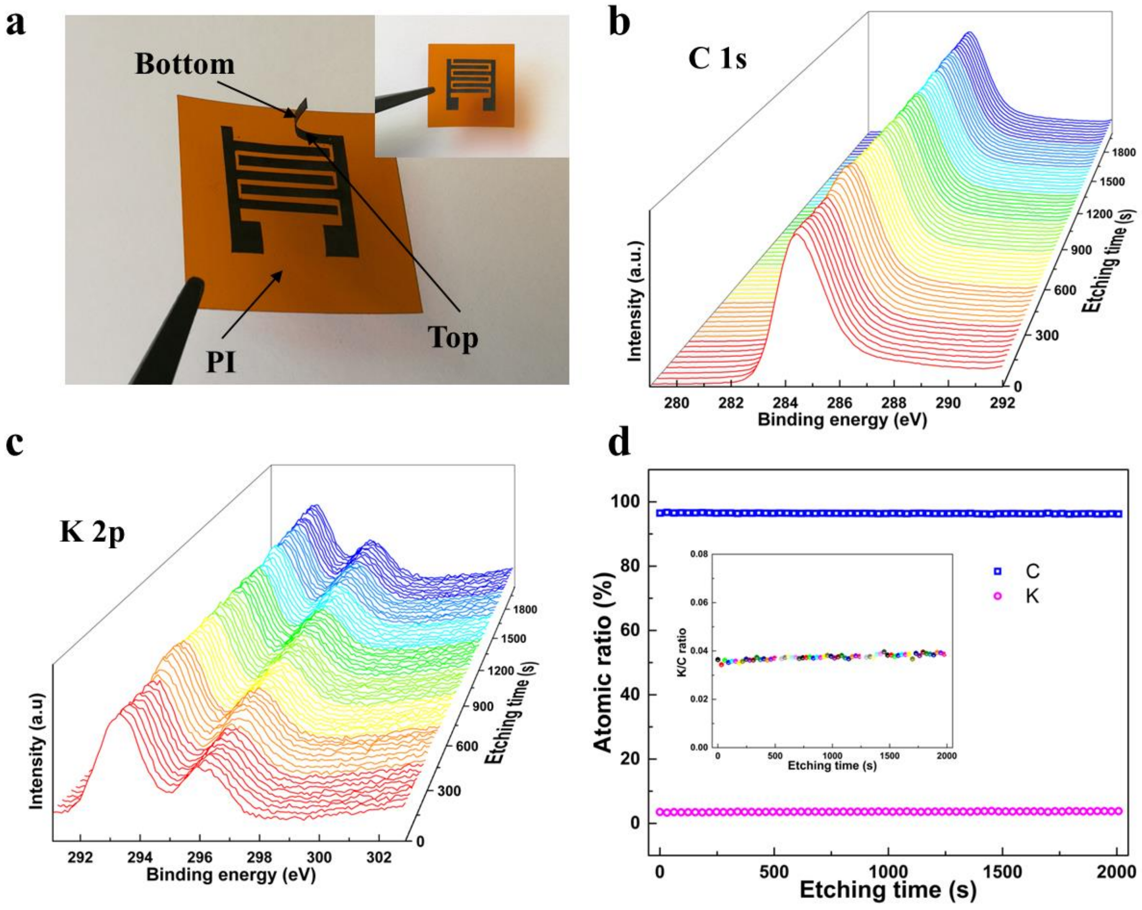

Figure S13. (a) Photograph of a graphene film on PI substrate; (b-c) C 1s and K 2p XPS depth profiling spectra of the graphene film electrode after cycling.

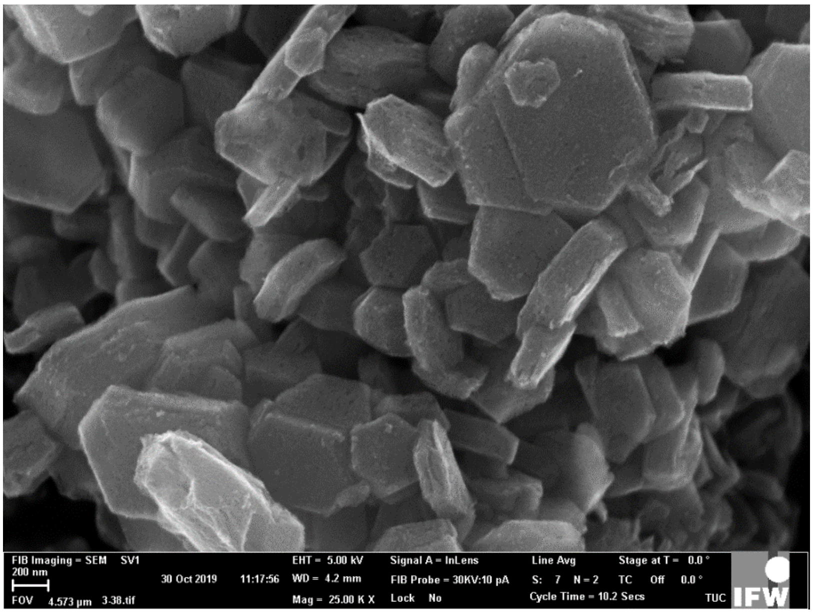

Figure S14. SEM image of the porous graphene after 10000 cycles.

Table S3. The charge transfer resistance (Rct) and the internal resistance (Rs) of the graphene-based MSC after different charge/discharge cycles.

Cycling number	Rs	Rct
1	264.2 Ω	77.3 Ω
20	264.5 Ω	77.6 Ω
500	273.1 Ω	90.9 Ω
1000	273.1 Ω	102.3 Ω
2000	274.2 Ω	111.4 Ω
8000	281.3 Ω	195.5 Ω
10000	281.6 Ω	209.1 Ω

Table S4. A detailed comparison of electrochemical performance of various graphene-based MSCs.

MSCs	Voltage	Capacitance	Cycling stability	Energy density	Power density	Ref.
CVD graphene films	0-1.0 V	3.8 mF cm^{-2}	96% (20000)	23 mWh cm^{-3}	1860 W cm^{-3}	*Adv. Mater.* **2018**, *30*, 1801384
rGO	0-0.8 V	462 μF cm^{-2}	90% (1000)	31.9 mWh cm^{-3}	324 W cm^{-3}	*Adv. Mater.* **2013**, *25*, 4035–4042
FG-MSC	0-3.5 V	14.2 mF cm^{-2}	93% (5000)	56 mWh cm^{-3}	21 W cm^{-3}	*J. Am. Chem. Soc.* **2018**, 140, 8198-8205
B-LIG-MSC	0-1.0 V	16.5 mF cm^{-2}	90% (12000)	0.52 mW h cm^{-3}	3.2 W cm^{-3}	*ACS Nano* **2015**, 9. 5868–5875
LIG-N-PEDOT	0-0.8 V	720 μF cm^{-2}	96% (10000)	64 μW h cm^{-2}	32.9 mW cm^{-2}	*Small* **2018**, *14*, 1702249
all-graphene MSC	0-1.0 V	313 μF cm^{-2}	65% (11000)	0.2 mW h cm^{-3}	4 mW cm^{-3}	*Nanoscale* **2019**, 11, 10172–10177
MPG-MSCs	0-1.0 V	17.9 F cm^{-3}	98.3% (100000)	2.5 mWh cm^{-3}	495 Wcm^{-3}	*Nat. Commun.* **2013**, 4, 2487
EG-MSs	0-0.8 V	7.6 mF cm^{-2}	91.1% (10000)	19.6 mWh cm^{-3}	2.8 W cm^{-3}	*Adv. Mater.* **2017**, *29*, 1703034
graphene	0-1.0 V	17.8 F cm^{-3}	100% (10000)	1.29 mWh cm^{-3}	278 W cm^{-3}	*Adv. Energy Mater.* **2016**, *6*, 1600909
FHG	0-1.0 V	6.41 mF cm^{-2}	88.6% (10000)	4.24 mW h cm^{-3}	0.12 W cm^{-3}	*J. Mater. Chem. A* **2019**, 7, 7852–7858
LSG	0-1.0 V	25.1 mF cm^{-2}	99.2% (12000)	0.657 mWh cm^{-3}	1000 mW cm^{-3}	*Adv. Energy Mater.* **2018**, *8*, 1801840
LPG-MPS	0-0.9 V	3.9 mF cm^{-2}	93% (20000)	0.98 mWh cm^{-3}	300 mW cm^{-3}	*Nano Energy* **2016**, 26, 276-285
LIG-MSCs	0-0.6 V	0.62 mF cm^{-2}	100% (10000)	0.92 μWh cm^{-2}	8 μW h cm^{-2}	*Adv. Funct. Mater.* **2019**, *29*, 1902860
graphene	0-1.0 V	0.7 mF cm^{-2}	77 % (11000)	1 mWh cm^{-3}	0.1 W cm^{-3}	*ACS Nano* **2017**, 11, 8249-8256
CVD graphene	0-1.0 V	36.7 F cm^{-3}	91.3% (10000)	5.1 mW h cm^{-3}	NA	*Nanoscal* **2017**, 9, 6998–7005
LSG-MSC	0-2.5 V	3.05 F cm^{-3}	100% (30000)	2 mW h cm^{-3}	200 W cm^{-3}	*Nat. Commun.* **2013**, 4, 1475
PG-MSCs	0-3.0 V	9.8 mF cm^{-2}	NA	11.6 mWh cm^{-3}	1500 mW cm^{-3}	*ACS Nano* **2017**, 11, 7284-7292
WJM graphene	0-1.8 V	5.3 mF cm^{-2}	98% (10000)	0.064 μWh cm^{-2}	20 mW cm^{-2}	*Adv. Funct. Mater.* **2019**, *29*, 1807659
EG	0-1.0 V	5.4 mF cm^{-2}	90% (5000)	NA	NA	*Adv. Mater.* **2016**, *28*, 2217–2222
Graphene	0-1.0 V	268 μF cm^{-2}	NA	NA	NA	*Adv. Energy Mater.* **2017**, *7*, 1700285
GQDs	0-2.7 V	468.1 μF cm^{-2}	97.8% (5000)	0.474 μWh cm^{-2}	56.7 μW cm^{-2}	*Adv. Funct. Mater.* **2013**, *23*, 4111–4122
3D GP	0-1.0 V	1.5 mF cm^{-2}	100% (20000)	0.38 μWh cm^{-2}	14.4 mW cm^{-2}	*small* **2017**, 13, 1603114
GTT-MSCs	0-1.0 V	27.2 mF cm^{-2}	98% (10000)	NA	1.57 mWh cm^{-3}	*Small* **2019**, *15*, 1901494
**graphene**	**0-1.4 V**	**21.7 mF cm^{-2} /10.9 F cm^{-3}**	**98.8% (10000)**	**5.0 μW h cm^{-2} /2.5 mW h cm^{-3}**	**6.0 mW cm^{-2} /3.0 W cm^{-3}**	**This work**

FG: fluorinated graphene

B-LIG: boron-doped laser-induced graphene

LIG-N-PEDOT: N-doped laser-induced graphene absorbing poly(3,4-ethylenedioxythiophene)

MPG film: a reduced graphene film

EG-MSs: ionic-liquid-based planar MSs based on pure electrochemically exfoliated graphene films

FHG: functionalized holey graphene

LSG: laser-scribed graphene

LPG-MPS: laser-processed graphene based micro-planar supercapacitor

LIG-MSCs: laserinduced graphene (LIG) for all-solid-state planar integrated MSCs

PG-MSCs: electrochemically exfoliated graphene in ionic liquid electrolyte

WJM graphene: single-/few-layer flakes of graphene based on high-pressure wet-jet-milling

EG: electrochemically exfoliated graphene

GQDs: graphene quantum dots

3D GP: three dimensional graphene pellets

GTT-MSCs: graphene- and the organic charge transfer compound tetrathiafulvalene-tetracyanoquinodimethane- based MSCs

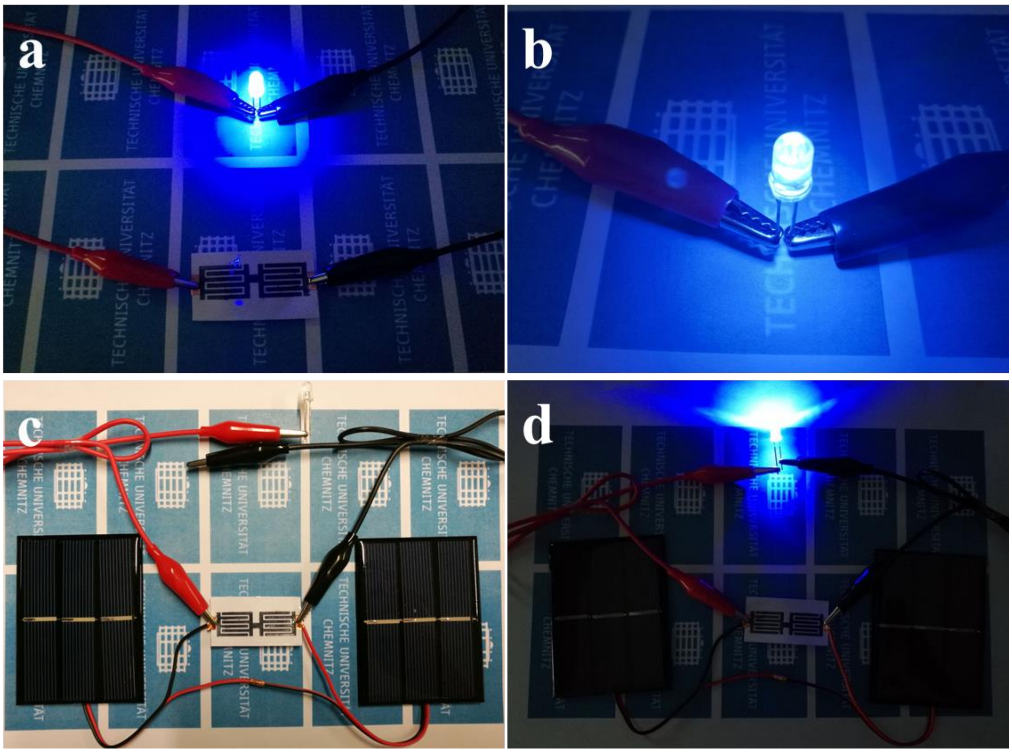

Figure S15. (a, b) Photographs of a LED powered by the MSCs in series; (c) Photographs of the practical circuit with MSCs, solar cells and a LED; (d) A lit LED driven by the energy stored in MSCs.
